# Mr. Barlow, on the Delivery of the Placenta

**Published:** 1800-10

**Authors:** James Barlow

**Affiliations:** Blackburn, Lancashire


### *To the Editors of the Medical and Physical Journal.*

Gentlemen,

On perusing your valuable periodical Work, I have observed a discordancy of opinion relative to the expediency of the extraction of the placenta, which has induced me to present you with a few observations on that subject.

Naturam expellas furca tamen usque recurret. Hor.

It will scarcely be thought necessary to trace the different opinions which have been advanced on this subject, from the remotest periods of antiquity to the present day; nor would a knowledge of the practice of the ante-diluvians, or even the method adopted by our first parents with regard to their children, illustrate the point in discussion; for as they were in a state of Nature, it is natural to conclude, (setting aside the interposition of Providence) that no manual assistance for the extraction of the placenta, at so early a period of time, was necessary; and if the human race had existed in a state of Nature to the present time, it is highly probable, there could have required no other aid for the extrusion of the placenta, than what was natural to every species of animal in their primordial state: and as the female of every animal in a state of parturition is possessed of a placenta, or substance analogous thereto, it might, on a superficial view, lead us to conclude, that no difficulty would arise from its retention amongst the brute creation, seeing they approach nearer to a state of Nature than the human; but, upon a more careful attention to their various stages of parturition, we shall find them labouring under certain difficulties, though, perhaps, in a less degree than what falls to the lot of woman.

It has been observed by various authors who have had opportunities of visiting the most uncivilized parts of the globe, where the inhabitants live in a great measure in a state of Nature, that the women suffer comparatively little with those of the European countries.*

The difference then attendant on human parturition is more ascribable to certain modes of living, and deprivations in society, than to any physical necessity, or curse announced by the

* Vide Long's History of Jamaica, Vol. II. page 380. "Their women are delivered with little or no labour; they have, therefore, no more occasion for midwifes than the female Oranoutang, or any other wild animal. A woman brings forth her child in a quarter of an hour, and goes the same day to the sea and washes herself."

the Deity to our firſt parents, as a puniſhment inflicted upon the fallen nature of man.*

There is no occaſion then to aſcribe evils to our firſt parents, which we daily bring upon ourſelves, nor blame the benevolent hand of Providence in cauſing us to go erect, though Dr. Oſborn, page 9th, ſays, the erect poſition of the human frame, that ſingular mark of pre-eminence, expoſes women to pain and difficulty in natural parturition, from which the ſubordinate quadruped is almoſt entirely exempted, by the horizontal poſition of her body. Surely ſuch an idea will never be generally credited; the cauſe muſt ariſe from circumſtances of a more adventitious nature, and not from any pre-eminence of poſture, or inevitable phyſical evil; even a ſuperficial view of the preſent ſtate of human nature will eaſily convince any one of the origin of this depravity.†

Whoever takes the trouble of collecting the various opinions advanced by authors on this ſubject, from the time of Hippocrates, will have reaſon to lament that this branch of midwifery ſtill remains a point unſettled amongſt accouchers; and as the event of the mother ſo often turns on a right or wrong mode of managing the delivery of the placenta, ſurely it will not be deemed an uſeleſs undertaking in me, to condenſe into a narrow compaſs the mode of practice I have hitherto adopted with ſucceſs.

On peruſing the books of Celſus, Ambroſe Parey, Mauriceau, Pugh, Deventer, Mawbray, Roederer, Aſtruc, Dionis, Burton, Chapman, Counſelo, Exton, Maningham, and Giffard, I find them recommending an early extraction of the placenta, ſome of whom adviſing the introduction of the hand, even before the diviſion of the funis, fearing the internal orifice of the womb ſhould cloſe and obſtruct the extraction; but this,

---

† Dr Oſborne ſeems to rely much on the Moſaic account, that "in ſorrow thou ſhalt bring forth children." If this were the caſe, it would be applicable, in an equal degree, to every parturient female in all quarters of the globe; but this we find does not take place, for it is not very uncommon in this country, to obſerve a labour terminate with little or no appearance of pain, nor the leaſt degree of ſorrow, but ſometimes even emotions of joy may be obſerved upon the countenance. And we read in Wafer's Deſcription of the Iſthmus of America, page 360, that, "When a woman is delivered of a child, another woman takes it in her arms, within half an hour or leſs after it is born, and takes the lying-in woman upon her back, and goes with both of them into the river and waſhes them there." The Brazilian women have very eaſy labours, and as ſoon as they are delivered, they go to the river and waſh themſelves without harm. Vide Newhoff's Voyages, p. 151.

† See Hufeland on the Art of Prolonging Life, Vol. II. page 9, chap. 1; and Dr. Gregory's Comparative View, ſect. 1.

this, amongſt many other abſurd cuſtoms, is now entirely exploded. But as it is natural to ſuppoſe, that each practitioner in midwifery is ſtrongly enliſted to the practice of thoſe from whom he has been inſtructed in the art, we need not be ſurpriſed at the diverſity of opinions which ſtill prevail, nor even wonder at the various ſucceſs attendant thereon. A thorough knowledge of the anatomy of the gravid uterus in every ſtage of parturition, is an indiſpenſible requiſite to every accoucheur, and will prove a barrier againſt a hoſt of difficulties and embarraſsments which frequently occur in practice.

There are two oppoſite prevailing opinions at this day, reſpecting the moſt advantageous mode of delivering the placenta; ſome adviſing, in all caſes, the manual extraction immediately after the birth of the child; others* purſue a different plan, making gentle efforts by pulling at the funis for ſome time; and if this fail, the extraction is then accompliſhed by manual aſſiſtance.

There are a few, alſo, who think no harm enſues from truſting its extruſion intirely to Nature in every inſtance. Were it poſſible from ſuch a diverſity of opinions, to adduce a true eſtimate of the advantages and diſadvantages attendant on theſe various modes of practice upon the moſt enlarged ſcale, I fear more harm than good would be found to reſult from ſuch a calculation, if contraſted with a ſimilar computation, and where the woman was totally left without the leaſt aſſiſtance.

Though the exit of the placenta, were it left entirely to Nature, would ſeldom require manual extraction, yet, as the powers of the uterus are often limited, and as urgent ſymptoms ſometimes occur, it will generally be acknowledged, that the aſſiſtance of art, in theſe caſes, becomes equally neceſſary as in any branch of ſurgery. Soon after the birth of the child, the uterus in part contracts upon the ſubſtance of the ſecundines, by which inherent power of contraction, aſſiſted by the action of the abdominal muſcles, the placenta is generally expelled naturally in the ſpace of ten or fifteen minutes. When the efforts of Nature are all ſufficient to accompliſh this work in the above-mentioned time, it will be uſeleſs to obtrude the hand into the uterus for the purpoſe of extracting the placenta, before having firſt had reaſon to doubt the ſufficiency of the expulſive efforts of that organ. If after waiting the time above ſpecified, and the uterus aſſumes no diſpoſition to diſlodge its contents,

* Amongſt whom are Smellie, Hamilton, Baudelocque, Johnſon, Denman, White, Foſter, and Perfect.

contents, it may then be proper, after placing the patient in a horizontal poſition, to introduce one or two fingers of one hand up the vagina uteri, whilſt with the other we take hold of the funis and make a gentle diſtenſion, by which means we ſhall be enabled to aſcertain the ſtate of the os uteri and ſituation of the placenta. If the mouth of that viſcus ſhould be found in a relaxed condition, and the placenta within reach of the finger, we need not deſpair of promoting its expulſion, by making a gentle preſſure upon the abdomen with one hand, and cautiouſly pull* at the funis with the other, in a direction with the axis of the pelvis; this mode of acting will generally ſtimulate the uterus, and ſeldom fail to ſolicit the expulſion of the placenta. But when no flooding, or other urgent ſymptoms occur, and when the extruſion of the placenta does not follow, we may with ſafety deſiſt from theſe gentle efforts, and wait even two, three, or more hours, before any further attempts are made for its extraction by artificial means.

When the placenta is thus detained, there is ſeldom any hæmorrhage during that interval; but when the pains recur, and the placenta becomes detached from the uterus, a degree of flooding will ſupervene, and our conduct muſt be regulated accordingly.

In caſes where the placenta is only in part detached from the uterus, we may naturally expect a leſs diſcharge to enſue, than where the whole of the placenta adheres to that viſcus; conſequently, it will be more prudent to wait for the natural excluſion of the placenta in the latter caſe than in the former; it, however, not unfrequently happens, that the uterine actions are irregular, as in the caſe of flooding and ſyncope, or after a tedious labour; in which caſe, our conduct muſt be governed by attendant circumſtances, and the quantity of blood loſt in a given time. I would not have it underſtood, that every diſcharge of blood from the uterus is a ſufficient juſtification for the immediate extraction of the placenta in every inſtance; ſometimes a diſcharge of *coagula* will take place, in which caſe it becomes neceſſary to aſcertain the quantity and quality of the diſcharge before manual aſſiſtance be attempted. An uterine hæmorrhage taking place immediately after the birth of the child, and continuing without intermiſſion to diſcharge florid blood from the mouth of the uterine veſſels which open into its cavity, is, in itſelf, ſufficiently alarming, and calls for immediate aſſiſtance by art.

It

* It is a rule with me, never to exert a greater force upon the funis for the purpoſe of extracting the placenta, than what is adequate to ſupport its own weight, leſt a rupture or laceration ſhould take place.

It sometimes happens, that only a circumscribed portion of the placenta is detached from the uterus, whilst the membranes adhering around may confine the blood for a certain time and prevent hæmorrhage.†

In every case requiring manual extraction, the separation of the placenta from the uterus should be undertaken with the greatest caution; a gentle pressure with one hand upon the hypogastric region, to steady the uterus, will much facilitate the extraction. If an hæmorrhage should supervene the exit of the placenta, it will be found of service to administer a glyster, composed of four ounces of cold water, and eighty drops of tinct. opii; or an astringent injection may be thrown into the uterus to moderate the discharge; and in cases when the whole or part of the placenta remains in the womb for some days after the delivery of the child, it may be proper to force injections of warm water, or Port wine, into the uterus from time to time, to bring away any putrid *coagula* that is lodged in the uterus or vagina; this may also assist in promoting the expulsive efforts of the uterus, and dislodge the placenta. The adhesion of the placenta will sometimes be so great as to resist these gentle attempts for its expulsion; in which case it becomes necessary for the accoucheur to determine whether manual extraction, or abandoning its exit wholly to the efforts of Nature, have a preference: In this case every practitioner must be guided by circumstances, as the putrefaction of the placenta, when retained in the cavity of the uterus, is certainly a matter of considerable moment; yet, whilst the whole of the placenta remains firmly attached to the uterus, that process can only take place slowly: But, on the contrary, when that redundant mass becomes putrefied, it naturally follows that a separation, or as it were an exfoliation takes place, and that vital organ, the uterus, discharges the dead mass. From hence will be perceived, how difficult it is to lay down any determinate rules to be invariably followed in every case, yet a prudent practitioner will seldom be at a loss how to act; it is however a desirable event, and what I should wish to inculcate, that the expulsion of the placenta should generally be urged to follow the delivery of the child in a longer or shorter time, according to the emergency of the case. The detention of the placenta sometimes arises from an atony of the uterus, or an irregularity of contraction of the muscular fibres, assuming various shapes; in some cases, the mouth of the uterus has been found contracted upon the funis soon after the birth of the child; in others

† Albinus mentions a case of this kind.

others it aſſumes the form of an hour-glaſs, and is contracted in the middle, and the placenta is lodged as it were in a cyſt beyond this ſecond entrance; theſe and other ſpaſmodic contractions have been obſerved by different authors, and ſeem more liable to affect ſome conſtitutions in a greater degree than others. Smellie enumerates ſeveral inſtances which ſufficiently illuſtrate this fact. Perfect gives an account of a lady, who ſuffered from a retention of the placenta in ſix ſucceeding labours, in ſome of which it was retained till the third, and in one as long as the fourth day, ſee Vol. i. p. 91; ſee alſo Vol. ii. of the ſame author, where are ſeveral caſes related of a ſimilar nature, p. 377, 379, 381, 383, 387, 390, 393. As it is a ſatisfaction to adduce the opinion of ſo eminent a teacher of Midwifery as the late Dr. Colin Mackenzie on this ſubject, I will tranſcribe his anſwer to the 137th caſe, p. 383 of Perfect's Midwifery, Vol. ii.

"Dear Sir,

"As a flooding preceded the delivery, dangerous conſequences might have been expected from forcibly extracting the placenta; it was a precipitate meaſure by which the hæmorrhage moſt probably was much increaſed: In ſuch caſes, I have often allowed an hour, ſometimes an hour and a half; and without the ſymptoms are urgent, this will always be found the ſafeſt way. It is an idle prejudice, which obtains in reſpect to the retention of the placenta; in moſt caſes it is much ſafer, that it ſhould entirely be left to Nature, than to riſque the dangers which attend its forcible extraction; and yet it is often very diſagreeable to ſubmit its excluſion to Nature; but it muſt be remembered, that in great force there is more than proportionable danger of doing injury to the uterus; the medium between theſe two extremes, if any, will perhaps be found the beſt guide to our practice."

"I am, &c."

Exton mentions a caſe of contracted uterus: "As ſoon," ſays he, "as I had ſeparated the child I immediately paſſed my hand, which is my conſtant practice, and found the mouth of the womb ſo ſtrongly contracted that I could by no means introduce even a finger into it. I endeavoured to open the os uteri by paſſing a finger; but the contraction was ſo very ſtrong, that I believe the mouth of the womb would ſooner have been broke than yielded to my fingers. By the feel, it was like a purſe ſtrongly drawn up. After waiting twenty-four hours, (he adds) I perceived that the os uteri was quite relaxed, and fetched the placenta whole, without any difficulty." See p. 111. The ſame author, ſpeaking of the ſpaſmodic contraction of the os uteri taking place instantaneously after the birth of the child, says, "This, I think, is the only case that forbids an immediate extraction of the placenta, because of the great danger there is of hurting the woman, by being obliged to make use of too much violence." See p. 134. At pages 137, 138, and 139, are three more cases of contracted uterus related.

Mr. White, in his Treatise on the Management of Pregnant and Lying-in Women, relates seven cases where the placenta was retained by a spasmodic affection of the uterus. See fifth edition, p. 443, Case 21, where mention is made of the placenta not being expelled till the seventh day after delivery, and the patient recovered. More cases of the same species are mentioned by the same author; see p. 305, 307, 308, 309, 310.

Dr. Johnson relates a case where the uterus was violently contracted, about mid-way between the orifice and fundus; yet, after much difficulty, he gained admission by introducing the hand, and extracted the placenta with safety to the mother. See p. 2 6.

La Motte has seven cases of this sort. Vide his Observations, p. 358, 359, 362, 363.

Giffard relates two cases of contracted uterus. See p. 263, case 107; and p. 303, case 127.

Burton mentions a case of spasmodic affection of the uterus, of the sand-glass form. See p. 132.

Chapman relates two successful cases of contracted uterus, in one of which the placenta was extracted after five days retension. See p. 158.

Dr. Denman makes mention of a case where the placenta was retained till the fifteenth day after the birth of the child; but is silent as to the event.

In a manuscript copy of a course of Lectures which I possess, from Dr. Young, of Edinburgh, there is related an instance of retained placenta from the same cause: "The woman had been two hours brought to bed, and all the different methods had been used in order to extract the placenta. The cord was broke; I put the woman upon her side, and introduced my hand, but could not get hold of the placenta; I got my hand up to it but no further, the uterus having formed a sort of pouch for it; so that at last I was obliged to trust the matter to Nature; and, what was very uncommon, no fœtid discharge followed, nor any thing like the placenta, and yet the woman recovered, and did very well."*

---

* A similar case is mentioned, and where no dangerous symptoms followed. See Nisbet's Clinical Guide, Part iii. p. 145.

I myfelf once witneffed the fpafmodic difpofition of the neck of the uterus in a great degree; immediately after the head of the fœtus was emerged at the valva, the os uteri encircled round the neck of the fœtus like a collar, infomuch that the fœtus was ftrangled, and it was impoffible to extract the body, or even to introduce one finger between the os uteri and neck of the child. I adminiftered a large dofe of tinct. opii, which counteracted the fpafm, and the labour was terminated, with fafety to the mother, in about the fpace of two hours from the firft appearance of the fpafm. Were it neceffary I could adduce more cafes of fpafmodic affection of the uterus; but it will be perceived, by referring to the above-mentioned authors, that their mode of practice was widely different, moft of whom ftrenuoufly recommend the introduction of the hand, though the os uteri fhould be nearly clofed upon the funis, rather than trufting its expulfion to Nature, or waiting for a removal of the fpafm, or even attempting to counteract its effects by antifpafmodic medicines. An accurate knowledge of the uterine action will, in general, direct the accoucheur to the beft refources; for, in cafes of this nature, manual extraction can only be juftifiable in preffing emergencies; and as every individual cafe attended with fpafm may not require the fame treatment in every inftance, it may then be expedient to vary our mode of conduct as occafion requires; and when the os uteri is fo far contracted as to render the introduction of the hand impracticable, and no hæmorrhage or untoward fymptoms occur, I would recommend, in the firft place, to attempt the removal of the fpafm by giving the patient a pill with two or three grains of opium; or, what I have more than once found of greater efficacy, a clyfter compofed of ninety drops of tinct. opii to four ounces of cold water and a drachm of affafœtida, after which the hand may in general be cautioufly and gently infinuated into the uterus, and the placenta extracted, taking care not to exert too much force upon the os uteri, left inflammation, or laceration of the neck of that vifcus, fhould enfue.† I am, neverthelefs, certain, from the mufcular fibres of the uterus being fo circuitoufly arranged, that it is thereby poffeffed of a greater degree of elafticity than moft other organs of the human body, and is perhaps alfo lefs fenfible to injury; this every accoucheur, who has had even a tolerable fhare of practice, muft have witneffed; yet who would

† Fielding Ould is of opinion that the orifice of the womb is not capable of fo fpeedy a contraction as is generally imagined, and endeavours to controvert the opinion of Deventer on the fubject.

attempt the immediate extraction of the placenta, where the uterus is affected with ſpaſm, and no hæmorrhage or other dangerous ſymptoms prevail, before the above means have been employed for its removal. Should the ſpaſm be accompanied with hæmorrhage (which is ſeldom the caſe) we may conclude that a portion at leaſt of the placenta is detached from the uterus, therefore our conduct muſt be guided by the quantity of the diſcharge and ſtrength of the patient; in which caſe we ſhall generally find the introduction of the hand into the uterus more eaſily admiſſible than if no hæmorrhage has accompanied the ſpaſm. If, after the extraction of the placenta is completed, the uterus ſhould remain in an atonic ſtate, ſome degree of hæmorrhage will unavoidably ſupervene, owing to the mouth of the uterine veſſels remaining in a patulous ſtate; and being replenished with blood from another ſource, the muſcular fibres not performing their regular functions, the mouth of the womb becomes in ſome caſes plugged up with coagulated blood, whilſt the uterus becomes diſtended with the effuſed blood to a conſiderable degree; a knowledge of its exiſtence may be aſcertained, either by an examination of the abdominal parieties, or, what is more certain, by the introduction of the hand into the cavity of the uterus; and, to prevent fever and inflammation, the preſence of the hand in that viſcus will be ready to ſcoop out the coagulated contents, and an aſtringent injection may be thrown into the cavity of the uterus; after which the ſwaithing of the abdomen may act as a preventive to a return of the effuſion of blood into the uterine cavity, and alſo ſupply that loſs of preſſure which the ſtate of geſtation had occaſioned. Some authors recommend the plugging of the vagina in this ſtate of torpor of the uterus; how far this may be of ſervice I cannot pretend to determine, as I have never put it in practice.

In caſes of oſſification or induration of the placenta, the preſence of which can only be identified by the introduction of the hand into the uterus, and as there will be a degree of reſiſtance of the contractile ſtate of the uterine fibres at the attached part of the indurated placenta, conſequently any force exerted upon this part for its ſeparation may occaſion hæmorrhage or laceration of that viſcus; hence will appear the advantage of removing only the detached portion of the placenta, and leaving all the morbid parts behind, to be diſcharged per vias naturales, or abſorbed into the ſyſtem by the lymphatics of the uterus. In every caſe, however, where the extraction of the placenta becomes neceſſary, and no morbid affection or other circumſtances occur to preclude its removal, I would urge a total extraction of the whole maſs, when it can be accompliſhed with ſafety to the mother, rather than leaving ing any fragments behind, which eventually produce flooding, inflammation, or fever.*

On the other hand, when the os uteri is closely contracted upon the placenta and funis, and several days have elapsed from the birth of the child, and no hæmorrhage ensues, I would caution the accoucheur against making any forcible attempts to introduce the hand before other means have been tried to abate the spasm, as the mouth of that organ will, in all probability, bear less irritation at this period than it otherwise would immediately after the birth of the child. I am,

GENTLEMEN,

Your humble servant,

JAMES BARLOW.

*Blackburn, Lancashire,*

*Aug.* 29, 1800.

* Pugh says, "Three times in my practice I have been sent for to women where the children had been delivered three days before, and one four days; and notwithstanding I extracted the placenta, and without using much force, that is, not so much as to either injure the womb or over fatigue my patient, two out of three died, and the other with very great difficulty escaped; and the only reason I can assign was from the putrefaction of the placenta, which was very great, and caused inflammation of the uterus, and brought on putrid fevers, which carried them off, the one on the fourth, and the other on the fifth day after the extraction." See p. 30. Vide also Heath's Baudelocque, Vol. ii. sect. 7.

